# Changes in serum ghrelin and resistin levels after sleeve gastrectomy versus one anastomosis gastric bypass: prospective cohort study

**DOI:** 10.1097/JS9.0000000000001608

**Published:** 2024-06-04

**Authors:** Fusun Ozmen, Tevfik T. Şahin, Anil Dolgun, M. Mahir Ozmen

**Affiliations:** a Department of Basic Oncology, Cancer Institute, Hacettepe University; b Depatment of Surgery, Medical School, Hacettepe University; c Department of Biostatistics, Medical School, Hacettepe University, Ankara; d Liver Transplant Institute, Inonu University, Malatya; e Department of Surgery, Faculty of Medicine, Bahcesehir University (BAU), Istanbul, Turkey; f Department of Surgery, Faculty of Medicine, University of La Sapienza, Rome, Italy

**Keywords:** gastric bypass, ghrelin, insulin resistance, obesity, one anastomosis, resistin, sleeve gastrectomy

## Abstract

**Introduction:**

Humoral factors and neural mechanisms play a central role in the pathogenesis of obesity and in weight loss following bariatric surgery. Although various hormones and adipokines, including ghrelin and resistin, are linked to obesity, studies analyzing the changes in fasting ghrelin and resistin levels in patients following one anastomosis gastric bypass (OAGB) are lacking.

**Aim:**

The authors aimed to investigate resistin and ghrelin levels before and after two commonly used bariatric procedures with different mechanisms of action: sleeve gastrectomy (SG) and OAGB.

**Patients and methods:**

Fasting serum ghrelin and resistin levels were evaluated by using ELISA in a nonrandomized, prospective cohort study for the pattern of changes in the preoperative period and 1 week, 1 month, 3 months and, 12 months after surgery in age and sex-matched patients with BMI ≥40 kg/m^2^ undergoing either SG (*n*=40) or OAGB (*n*=40). Their relationships with demographic parameters such as body weight, BMI, presence of T2DM, HbA_1_C, and Homeostatic Model Assessment for Insulin Resistance (HOMA-IR) index were also evaluated.

**Results:**

OAGB was superior in weight control compared to the SG group. There were significant differences in resistin and ghrelin levels between the OAGB and SG groups. Ghrelin decreased more in the SG group than the preoperative values. This change in ghrelin levels was more significant at 1 year after SG [preoperative mean (range) level of 334.2 (36.6–972.1) pg/ml decreased to 84 (9.1–227) pg/ml at 1 year] whereas in the OAGB group no significant change was observed [preoperative mean (range) level of 310 (146–548) pg/ml decreased to 264 (112–418) pg/ml at 1 year]. Resistin levels decreased in both groups, especially after 3 months and onward following both operations [the mean (range) resistin levels were 2.6 (0.87–5.4) ng/ml and decreased to 1.1 (0.5–2.4) ng/ml in the SG group vs 2.48 (0.89–6.43) ng/ml decreased to 0.72 (0.35–1.8) ng/ml in OAGB group at 1 year], which was in parallel with changes in HOMA-IR index, body weight, and BMI changes at 1st year. HOMA-IR index changes were similar, but more prominent after OAGB. OAGB was als3 three months and onward), and HOMA-IR changes.

**Conclusion:**

This is the first study to compare fasting ghrelin and resistin levels after OAGB and SG. Although similar changes were observed, ghrelin changes were more prominent after SG, whereas resistin were observed after OAGB. OAGB was superior in T2DM control, which was in parallel with weight loss, fasting resistin levels, and HOMA-IR changes suggesting a possible effect of resistin after OAGB in glucose metabolism and insulin resistance.

## Introduction

Highlights

- This nonrandomized, prospective cohort study investigates fasting ghrelin and resistin levels in patients undergoing either restrictive [sleeve gastrectomy (SG)] or malabsorptive [one anastomosis gastric bypass (OAGB)] procedures for morbid obesity before and after (1 week, 1 month, 3 months, and 1 year) surgery.
- Fasting ghrelin levels decreased significantly after SG, and it was most prominent in 1st year compared to OAGB. Although resistin decrease was not significant in the first 3 months, it was evident at 1 year and was directly related to weight loss and Homeostatic Model Assessment for Insulin Resistance (HOMA-IR) index in, being more significant in the OAGB group.
- OAGB was more effective in the remission of type 2 diabetes, in parallel with weight loss, fasting resistin levels, and HOMA-IR changes.
- OAGB has better effects on weight loss and resolution of type 2 diabetes than SG.
- Although similar changes were observed in the fasting levels of resistin and ghrelin, ghrelin changes were more prominent after SG, whereas resistin changes were more prominent after OAGB.
- OAGB was superior in the control of T2DM, which was in parallel with weight loss, fasting resistin levels, and HOMA-IR changes, suggesting a possible effect of resistin on glucose metabolism and insulin resistance.

Obesity is a modern pandemic and is associated with various systemic diseases, among which diabetes mellitus is the most prominent. Although there are various treatment options for obesity and associated diseases, nonsurgical options are mainly used either as an initial treatment strategy or for weight gain, as they have marginal success. Bariatric surgery is currently accepted as the gold standard for the treatment of morbid obesity and individuals with systemic diseases associated with obesity.

Humoral factors and neural mechanisms play a central role in the pathogenesis of obesity and mechanisms of weight loss following bariatric surgery. Although various hormones and adipokines are linked to obesity, ghrelin and resistin are two molecules being actively investigated for possible roles in the mechanism of obesity^1–4^. Studies analyzing the changes in fasting ghrelin and resistin levels following one anastomosis gastric bypass (OAGB) are lacking.

Ghrelin is a 28 amino acids peptide gut hormone with an orexigenic role and specifically modulates food intake and lipid and glucose metabolism^5^. Ghrelin is released in response to the growth hormone secretagogue receptor (GHS-R) within the anterior lobe of the pituitary gland, which also influences food intake^5^. Ghrelin is synthesized and secreted primarily in the stomach and pancreas. In addition, it is secreted in lesser amounts from the kidneys, lungs, pituitary gland, and hypothalamus. It is present in plasma and saliva at ~100–200 pg/ml^6–8^.

Although it has already been shown that circulating ghrelin levels increase before meals (and during fasting), which stimulates the urge for food intake, and its levels decrease after food intake^9–11^, hormonal, and neural mechanisms responsible for ghrelin response are not fully understood and require clarification. This is especially true for the changes in its levels following various bariatric procedures.

On the other hand, it should also be noted that the results of the studies on the effects of glucose, protein, and fatty acids on gastric ghrelin secretions are also conflicting, mainly due to the design of the studies^12–15^.

Resistin is a 12.5-kDa peptide with 108 amino acids. Its plasma concentration ranges from 2.5–21.5 ng/ml. It is mainly produced by the adipose tissue. Resistin levels are increased in obese individuals^1^. In humans, resistin is also produced almost everywhere, including peripheral blood mononuclear cells, macrophages, and bone marrow cells, which are the primary sources of circulating resistin. In addition, resistin is produced to a lesser extent from the gastrointestinal cells to the hypothalamus^16–18^. Resistin inhibits cellular glucose uptake, which is related to an increases in triglycerides and cholesterol levels^17^. Resistin levels increase insulin resistance and dyslipidemia^19^. To the best of our knowledge, no study has investigated the changes in serum resistin levels following OAGB.

The present study aimed to investigate the fasting levels of resistin and ghrelin following two commonly used bariatric procedures: sleeve gastrectomy (SG) and OAGB. In addition, the relationships between these adipokines and the demographic parameters such as body weight, BMI, presence of T2DM, HbA1C, and Homeostatic Model Assessment for Insulin Resistance (HOMA-IR) index were analyzed.

## Patients and methods

### Study design

This was a nonrandomized, prospective cohort study conducted in patients undergoing obesity surgery.

Fasting serum ghrelin and resistin levels were evaluated prospectively for the pattern of changes in the preoperative period and 1st week, 1st, 3rd, and 12th months after surgery in patients undergoing either SG or OAGB.

All procedures were performed by the same surgical team, and SG was performed as explained by Ozmen and Gagner^20,21^. OAGB was performed according to the technique defined by Rutledge with a biliopancreatic limb length of 200 cm^22–25^.

This study was conducted in accordance with the standards set in the Declaration of Helsinki. The study protocol was approved by the Ethics Committee of Hacettepe University (GO 14/203-18), Ankara-Turkey and written informed consent was obtained from all participants. This study was supported by the Turkish Scientific and Research Council (TUBITAK) through a grant (B.14.2.TBT.0.06.03.02-161-195358).

**Figure 1:**
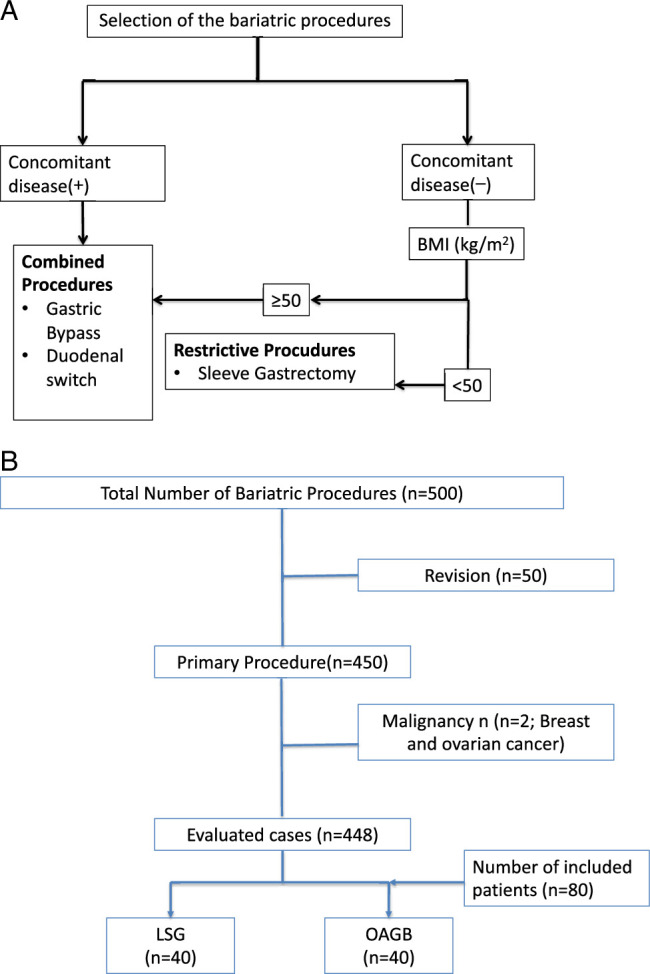
The summary of the type of the bariatric procedures and the selection criteria. (A). The selection of surgical procedures tailored according to the characteristics of the patients. (B). The flow chart of the patients that are included in the study.

The selection of the appropriate surgical procedure for the patients is summarized in Figure 1A. A flowchart of the total number of patients and the patients included in the study is outlined in Figure 1B.

### Patient selection

From the 500 bariatric operations performed during the study period (SG/OAGB: 298/202), 80 age and sex-matched primary bariatric cases with BMI ≥40 kg/m^2^ (SG/OAGB: 40/40) were included in the study, and serum ghrelin and resistin levels were evaluated prospectively for the pattern of changes in the preoperative and postoperative 1st week, 1st, 3rd and 12th months period alongside age, sex, BMI [body weight (kilograms)/height (meters)^2^], body weight (kg), HbA*1*C, HOMA-IR index (mg/dl) [HOMA-IR=Glucose (mg/dl) × Insulin (mIU/l)/405] and total weight loss (Table 1). The correlation between the demographic parameters and serum ghrelin and resistin levels were evaluated.

**Table 1:** The demographic data of the patients in the study groups.

	LSG (*n*=40)	OAGB (*n*=40)
Age (years)	35.2 (19–60)	38.2 (22–65)
Gender (Male/Female)	6/34	7/33
No with T2DM	4 (20%)	20 (50%)
Weight (kg)		
Preoperative	117.3 (93–144)	133.2 (102–193)
1^st^ week	108.6 (85–135)	122 (95–183)
1^st^ month	102 (79–128)	113 (86–172)
3^rd^ month	95.5 (71–120)	105 (80–161)
1^st^ year	78 (52–100)	80 (64–115)
BMI (kg/m^2^)		
Preoperative	43.3 (40–53.2)	49 (40.6–66.9)
1^st^ week	40 (37–50)	45 (38.5–63)
1^st^ month	38 (34–47)	42 (35–60)
3^rd^ month	35.5 (31–44)	40 (32–56)
1^st^ year	29 (23–37)	30 (26–40)
HbA1C (%)		
Preoperative	6 (5.0–6.8)	8.3 (5.2–10.7)
1^st^ week	5.9 (4.8–6.5)	7.9 (4.8–9.9)
1^st^ month	5.5 (4.8–6.2)	7.0 (4.7–8.4)
3^rd^ month	5.2 (4.7–5.9)	5.8 (4.5–7.1)
1^st^ year	5.0 (4.2–5.6)	5.3 (4.3–6.0)
HOMA-IR (mg/dl)		
Preoperative	3.7 (1.2–5.3)	5.9 (1.8–9.1)
1^st^ week	3.4 (1.0–5.0)	5.2 (1.0–8.6)
1^st^ month	2.5 (1.0–4.0)	4.0 (0.9–6.5)
3^rd^ month	1.6 (0.8–2.9)	2.7 (0.8–5.3)
1^st^ year	0.9 (0.6–1.3)	1.3 (0.6–2.2)
No with T2D (%)		
Preoperative	4 (20%)	20 (50%)
1^st^ week	4 (20%)	12 (30%)
1^st^ month	1 (2.5%)	6 (15%)
3^rd^ month	1 (2.5%)	3 (7.5%)
1^st^ year	1 (2.5%)	1 (2.5%)
Ghrelin (pg/ml)		
Preoperative	334 (36.6–972)	310 (146–548)
1^st^ week	204.8 (36–356)	251 (57–548)
1^st^ month	189 (30–472.3)	276 (56–526)
3^rd^ month	215 (30–348)	327 (127–964)
1^st^ year	84 (9–227)	264 (112–418)
Resistin (ng/ml)		
Preoperative	2.6078 (0.8618–5.389)	2.473 (0.892–6.426)
1^st^ week	3.5845 (1.069–8.079)	3.098 (1.016–6.028)
1^st^ month	2.6039 (0.7125–5.699)	2.541 (1.015–5.720)
3^rd^ month	2.2362 (0.990–5.402)	2.844 (0.790–8.482)
1^st^ year	1.128 (0.493–2.348)	0.723 (0.352–1.768)

All values were shown as mean (range).

Patients with previous abdominal surgery, including bariatric procedures, with malignancy, type 1 diabetes, liver and kidney problems, patients who use any medications for weight loss, and patients who developed complications during and after the operation were excluded from the study.

### Surgical techniques

All patients were informed about the risks and benefits of the surgery, and all provided written informed consent. Both procedures were laparoscopically performed by the same surgical team. The surgical techniques used in this study were SG and OAGB.

Vertical SG is performed using 36F bougie, transection of the stomach carried out by starting 2–4 cm from the pylorus and special attention is paid to remove the whole fundus by staying 1 cm away from the gastroesophageal junction^20,21^.

During OAGB procedure is mainly aimed at converting the stomach into a longer and narrower tube with an average length of 18–24 cm, extending from the angular incisura of the lesser curvature up to the angle of His. After gastrotomy, a gastroenterostomy around 200 cm distal to the ligament of Treitz was created, and the opening was closed using continuous sutures^22,23^.

### Postoperative management and follow-up

In our routine practice, SG patients do not have a nasogastric tube, whereas the nasogastric tube is removed on postoperative day one in gastric bypass patients. Upper gastrointestinal radiograph examination with orally ingested water-soluble contrast material was routinely performed postoperatively either on the first (for SG) or second day (for OAGB), and if the examination was normal, patients were started on a clear liquid diet, and the drain was removed. A low-calorie, protein-rich liquid, and mashed food diet was maintained during the first month. Multivitamins, vitamin D3, calcium, and iron were routinely prescribed to all the patients. Follow-up hospital clinical and complete biochemical evaluation visits were scheduled at 1, 3, 9, and 12 months after surgery.

### Sample collection and preparation for assay

Venous blood samples were collected from the patients into yellow-capped, gel vacuum biochemistry tubes after 8 h of fasting before blood collection. After 30 min of waiting for clotting, the collected blood samples were centrifuged at 2000×g for 10 min to separate the serum. The separated serum was dispensed into microcentrifuge tubes at a volume of 500 µl, and these tubes were stored at −80°C until the analysis by ELISA.

### ELISA (Enzyme Linked ImmunoSorbent Assay)

A human ghrelin ELISA kit (EZGRT-89K) (MilliporeSigma) was used to measure total ghrelin levels, and the sandwich ELISA procedure was carried out according to the manufacturer’s instructions with an intra-assay variation of <2% and interassay variation of <8%^26^.

A human resistin ELISA kit (ab183364) (Abcam) was used for the resistin assay, and the sandwich ELISA procedure was carried out according to the manufacturer’s instructions. The sensitivity of the assay was 24 pg/ml and the lowest detectable level of resistin was 78.1 pg/ml. The intra-assay and interassay coefficients of variation are 3 and 6%, respectively^27^.

### Statistical analysis

Data were recorded and analyzed using Statistical Software Package for Social Sciences (SPSS) for Mac version 22 (IBM). Independent samples *t*-test and Pearson correlation coefficient were used to determine the significance between relationships and comparisons of the hormone levels. Differences among groups were compared using one-way ANOVA, and the least significant differences were used to compare means among hormonal levels, BMI, body weight, HOMA-IR, and HbA_1_C. All values were shown as mean (range), and *P*<0.05 was taken as significant.

This work has been reported in line with the strengthening the reporting of cohort, cross-sectional, and case–control studies in surgery (STROCSS) criteria^28^.

## Results

### Laparoscopic sleeve gastrectomy (LSG) group

The mean age of the patients in the LSG group (*n*=40, 6 males/34 females) was 35.2 (19–60) years. Four (10%) patients had T2DM, and all were on oral antidiabetic drugs (OADs) only (Table 1).

**Figure 2:**
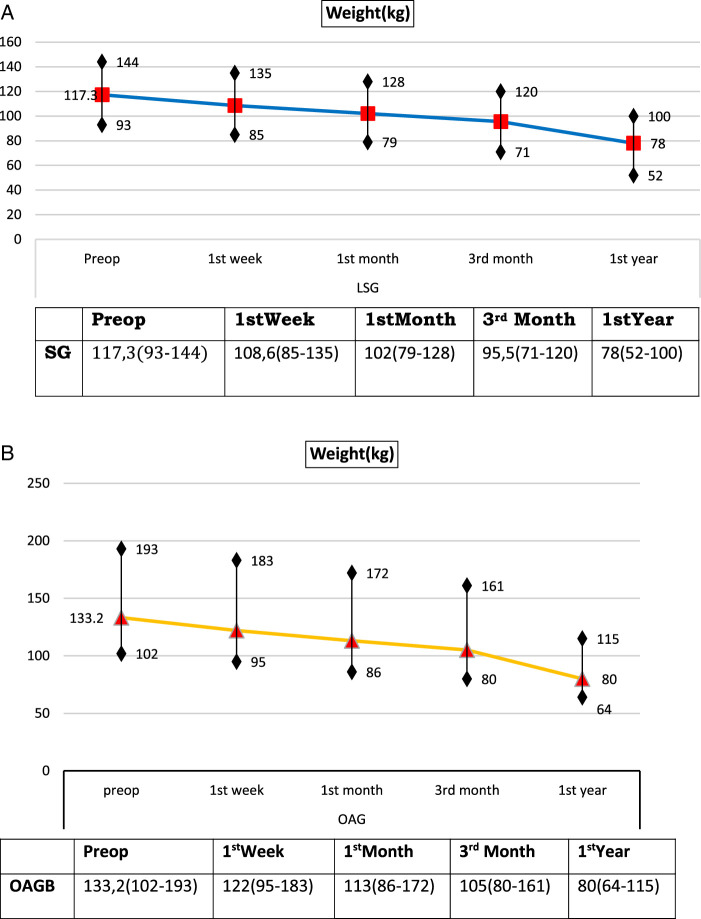
Changes on Weight (kg) (A) after LSG and (B) after LOAGB.

The preoperative mean (range) weight was 117.3 (93–144) kg. The postoperative mean (range) weight was 108.6 (85–135) kg, 102 (79–128) kg, 95.5 (71–120) kg, and 78 (52–100) kg at 1 week and 1, 3, and 12 months postoperatively, respectively (Table 1 and Fig. 2A).

**Figure 3:**
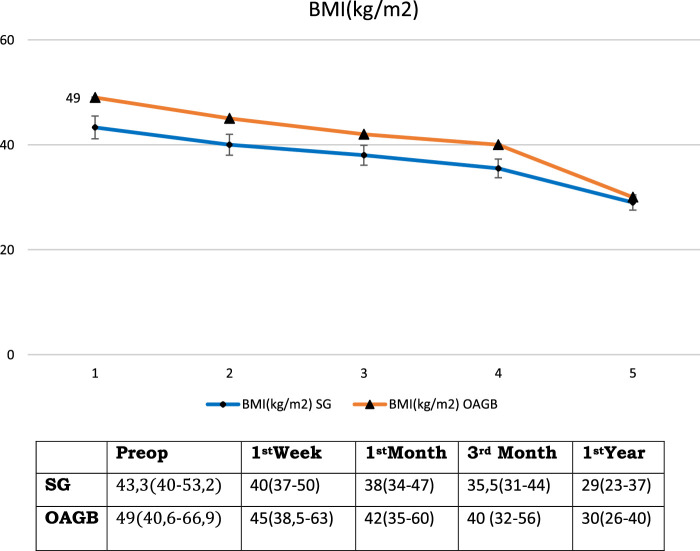
Changes on BMI (kg/m^2^).

The mean (range) BMI was 43.3 (40–53) kg/m^2^. BMI decreased to 40 (37–50) kg/m^2^, 38 (34–47) kg/m^2^, 35.5 (31–44) kg/m^2^, and 29 (23–37) kg/m^2^ at 1 week, 1 month, 3 months, and 12 months after surgery, respectively (Table 1 and Fig. 3).

**Figure 4:**
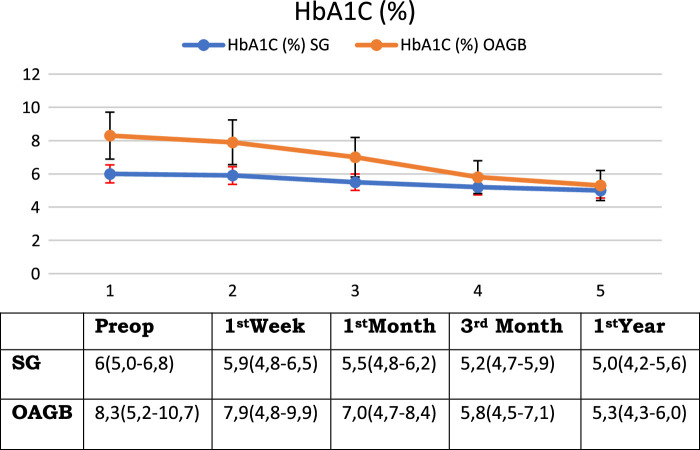
HbA1C levels before and after bariatric surgery.

The mean (range) HbA1C levels (normal range 4.7–5.6) were 6 (5.0–6.8) preoperatively and changed to 5.9 (4.8–6.5), 5.5 (4.8–6.2), 5.2 (4.7–5.9), and 5.0 (4.2–5.6) at postoperative 1 week, 1 month, 3 months, and 1 year, respectively (Table 1 and Fig. 4).

**Figure 5:**
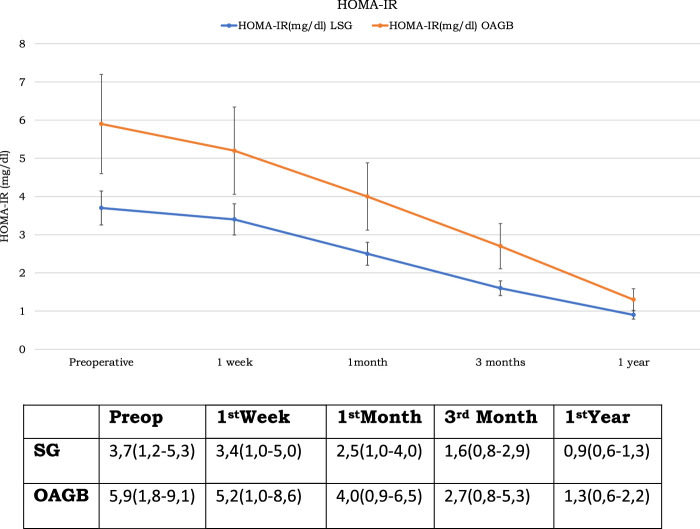
HOMA-IR levels before and after bariatric surgery.

The preoperative mean (range) HOMA-IR index were 3.7 (1.2–5.3) mg/dl and changed to 3.4 (1.0–5.0) mg/dl at 1st week, 2.5 (1.0–4.0 )mg/dl at 1st month, 1.6 (0.8–2.9) mg/dl at 3rd month and 0.9 (0.6–1.3) mg/dl at 1 year postoperatively (*P*<0.01 at 1, 3, and 12 months when compared to preoperative value) (Table 1 and Fig. 5).

**Figure 6:**
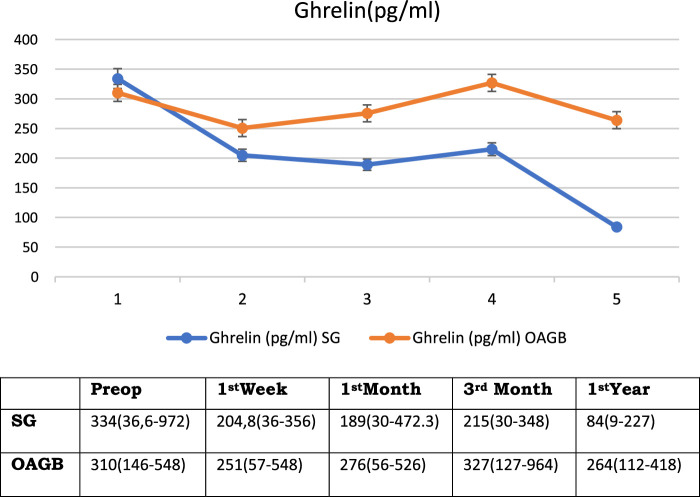
Fasting Ghrelin levels before and after bariatric surgery.

The mean (range) ghrelin levels were 334.2 (36.6–972.1) pg/ml, 204.8 (36.4–356.3) pg/ml, 188.9 (30–472.3) pg/ml, 225.1 (30.2–347.7) pg/ml, and 84 (9.1–227) pg/ml in the preoperative period, postoperative 1st week, 1st month, 3rd months and 12th months, respectively (*P<*0.01, when all periods are compared with each other) (Table 1 and Fig. 6).

**Figure 7:**
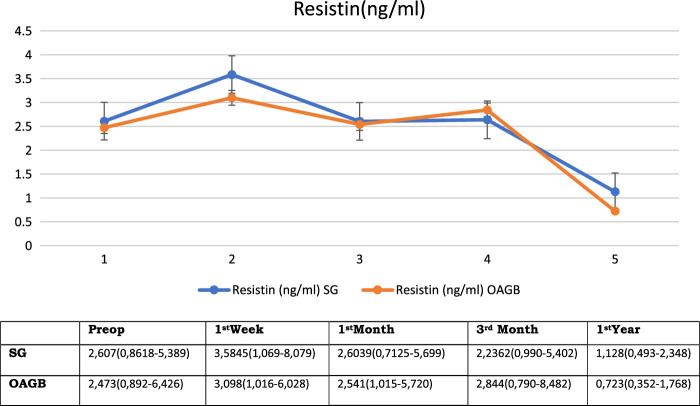
Fasting Resistin levels before and after bariatric surgery

The mean (range) resistin levels were 2.6078 (0.8618–5.389) ng/ml, 3.5845 (1.069–8.079) ng/ml, 2.6039 (0.7125–5.699) ng/ml, 2.6362 (0.990–5.402) ng/ml, and 1.128 (0.493–2.348) ng/ml in the preoperative period, postoperative periods 1st week, 1st month, 3rd months and 12th months, respectively (*P<*0.05, when all periods are compared with each other) (Table 1 and Fig. 7).

### Laparoscopic one anastomosis gastric bypass (LOAGB) group

The mean (range) age of the patients in the OAGB group (*n*=40, 7 males/33 females) was 38.2 (22–65) years. There were 20 (50%) patients had T2DM, 12 were on OADs, and 8 were on insulin treatment (Table 1).

The preoperative mean (range) weight was 133.2 (102–193) kg and the postoperative weight was 122 (95–183) kg, 113 (86–172) kg, 105 (80–161) kg, and 80 (64–115) kg at the postoperative 1st week, 1st, 3rd and 12th months, respectively (Table 1 and Fig. 2B).

The mean (range) BMI was 49 (41–67) kg/m^2^, which decreased to 45 (38.5–63) kg/m^2^, 42 (35–60) kg/m^2^, 40 (32–56) kg/m^2^, and 30 (26–40) kg/m^2^ in the postoperative 1st week, 1st, 3rd and 12th months, respectively (Table 1 and Fig. 3).

The mean (range) HbA1C levels were 8.3% (5.2–10.7%) preoperatively and changed to 7.9% (4.8–9.9%) 1 week after surgery. Further decreases were seen at 1 month to 7.0% (4.7–8.4%), which was followed by 5.8% (4.5–7.1%) and 5.3% (4.3–6.0%) at 3 months and 1st year, respectively (Table 1 and Fig. 4).

The preoperative mean (range) HOMA-IR index was 5.9 (1.8–9.1)mg/dl and changed to 5.2 (1.0–8.6) mg/dl at 1st week, 4.0 (0.9–6.5) mg/dl at 1st month, 2.7 (0.8–5.3) mg/dl at 3rd month and 1.3 (0.6–2.2)mg/dl at 1 year postoperatively (Table 1 and Fig. 5) (*P*<0.01 at all periods when compared to the preoperative values).

The mean (range) ghrelin levels were 310 (146–548) pg/ml, 251 (57–488) pg/ml, 267 (56–526) pg/ml, 327 (127–964) pg/ml, and 264 (112–418) pg/ml in the preoperative, postoperative 1st week, 1st, 3rd and 12th months period, respectively (*P<*0.05, when all periods are compared with each other) (Table 1 and Fig. 6).

The mean (range) resistin levels were 2.473 (0.892–6.426) ng/ml, 3.098 (1.016–6.028) ng/ml, 2.541 (1.015–5.720) ng/ml, 2.844 (0.790–8.482) ng/ml, and 0.723 (0.352–1.768) ng/ml in the preoperative, postoperative 1st week, 1st month, 3rd months and 12th months periods, respectively (*P*<0.05, when all periods are compared with each other) (Table 1 and Fig. 7).

There was a significant difference in terms of changes in resistin and ghrelin levels between the LOAGB and LSG groups (*P<*0.01). Ghrelin decreased more prominently in the SG group than in the preoperative values (*P*<0.05, when all periods are compared with each other). This change in ghrelin levels was more significant in 1st year after SG (*P*<0.01), whereas in the OAGB group, no significant change was observed. HOMA-IR index changes were similar, but were more prominent after OAGB, although there were more patients with diabetes. In the first year after surgery, there was still one patient (1/4, 25%) on OAD after SG and one patient (1/20, 5%) with 95% success in remission after OAGB. OAGB had a better success rate for T2DM (95%) in 1 year, even though patients with more severe diabetes underwent OAGB (40% of the patients were on insulin in the OAGB group vs none in the SG group) (Table 1). OAGB was superior in T2DM control, parallel with weight loss, fasting resistin levels (especially after 3 months), and HOMA-IR changes.

OAGB was superior to LSG in terms of weight control (*P*<0.05). Resistin levels decreased more in both groups in the postoperative 1st year compared to preoperative values and the first 3 months after both operations, which was in parallel with changes in HOMA-IR index and body weight and BMI changes in the 1st year.

## Discussion

The present study investigated fasting ghrelin and resistin levels in patients undergoing either restrictive (SG) or malabsorptive (OAGB) procedures for morbid obesity before and after (1 week, 1 month, 3 months, and 1 year) surgery. The correlation between demographic parameters such as body weight, BMI, presence of T2DM, HbA1C, HOMA-IR index, and fasting hormone (resistin and ghrelin) levels was also analyzed. We found that fasting ghrelin levels decreased significantly after SG, and it was most prominent in 1st year compared to OAGB. Although resistin decrease was not significant in the first 3 months, it was evident at 1 year and was directly related to weight loss and HOMA-IR index in both groups, being more significant in the OAGB group. OAGB was more effective in the remission of type 2 diabetes, in parallel with weight loss, fasting resistin levels, and HOMA-IR changes.

Ghrelin is the only gut hormone that induces appetite and energy intake by initiating alimentation; therefore, ghrelin levels increase during fasting and are suppressed by feeding. In normal subjects, serum ghrelin levels rise before meals and fall after ingestion during the day^28,29^. It is also found to be increased in patients on a diet that, in the end, prevents effective weight loss^30^. Ghrelin levels in normal subjects were also found to be inversely correlated with BMI, increase with hunger, and decrease after bariatric surgery^31,32^. In addition to its negative correlation with BMI, ghrelin levels are further lowered by insulin resistance and diabetes^33–36^.

However, there is some confusion regarding ghrelin levels after bariatric surgery in the literature, and the evidence is inconsistent as they measured the hormones at different times after surgery or measured the different forms of hormones^36–39^.

We demonstrated that fasting ghrelin levels started to decrease 1 week after SG, which was more prominent at 1 year and correlated with BMI. Our findings are consistent with those of previous studies, as summarized by McCarty *et al*.^40^ Ghrelin levels have also been shown to decrease^41–43^.

Despite the decrease in levels after surgery, the changes or the correlation with BMI were not significant at 3 months and one year after OAGB, which is also in agreement with a previous meta-analysis by Xu *et al*.^44^, even though they looked for Roux-en-Y gastric bypass. Skuratovskaia also reported similar changes in total ghrelin after SG and Roux-en-Y gastric bypass^32,44^.

This might be partly explained by the removal of the fundus during SG and the measured form of ghrelin, which was total fasting ghrelin, in our study.

Ghrelin may be in two forms: acyl-forms and deacyl-forms. In a meta-analysis by Wang *et al*.^33^, lower baseline levels of both ghrelin types were found. It was also shown previously that deacylated ghrelin levels are decreased with BMI, whereas acylated ghrelin levels are elevated with increased BMI^34^. Although total ghrelin levels do not seem to change after gastric bypass in 1 year, this might be due to the different response levels of different forms of ghrelin during surgery, especially after gastric bypass. A recent study by Bandt *et al*.^45^ revealed similar levels of fasting ghrelin two years after both OAGB and Roux-en-Y gastric bypass, although they did not have these levels before surgery.

Changes in ghrelin levels after Roux-n-y gastric bypass with and without fundus resection were evaluated, and it was found that fasting ghrelin levels were elevated 6 months and 1 year after surgery. However, this difference is not statistically significant^46,47^. There was also no significant difference in ghrelin levels between Roux-n-Y groups with and without fundus resection although it was much lower in patients with fundus resection, which supports our findings of decreased fasting ghrelin levels after SG as compared to OAGB resulting from the removal of most of the ghrelin-secreting cells during SG. Ghrelin levels positively correlated with weight loss, BMI changes, and HOMA-IR levels.

Obesity results from fat accumulation in the body, and adipose tissue is known to act as an endocrine organ that secretes some cytokines, adipokines, and resistin^15^. Resistin is a cysteine-rich protein that plays a role in inflammation, insulin resistance, and diabetes^48^.

A meta-analysis by Su *et al*.^48^ revealed that resistin levels were positively correlated with insulin resistance in obese individuals. Although it is not within the scope of this article, high plasma levels of resistin were also found to be correlated with proatherogenic inflammatory markers^49^, increased cardiovascular risk, unstable angina, and metabolic syndrome^50–52^. Studies examining resistin levels were controversial^53^. Marantos *et al*.^54^ found a decrease in resistin levels 12 months after surgery, but failed to show any correlation between anthropometric and metabolic parameters and resistin after SG, whereas a significant correlation was shown between resistin decrease and weight loss by Farey *et al*.^55^. We found the decrease in resistin levels after SG and it was in correlated to weight loss and HOMA-IR especially after 1 year.

The results of the studies after Roux-y gastric bypass are also conflicting. In their study, Caparros *et al*.^56^ showed that there was no difference in resistin levels between morbidly obese and lean subjects or between obese patients before and after gastric bypass surgery. Auguet *et al*.^57^, found that baseline resistin levels were higher in obese subjects and decreased 1 year after Roux-n-y gastric bypass surgery.

In their study, Sebunova *et al*. found a tendency to decrease but failed to show any statistically significant decrease 1 year after bariatric surgery, including both SG and Roux-n-y gastric bypass, and the decrease was more potent after Roux-n-y gastric bypass. They showed a strong correlation between resistin and weight, BMI, glucose, and HbA1C^58^.

To the best of our knowledge, the present study is the only study looking for ghrelin and resistin 1 year after OAGB in the English literature. Although similar changes were observed both after sleeve and OAGB 1 week, 1 month, and 3 months after surgery, the decrease in resistin levels was more prominent 1 year after sleeve or gastric bypass. The decreases at 1 year were in correlated with weight loss, BMI change, HbA1C, and HOMA-IR in both groups, suggesting a possible effect of resistin on glucose metabolism and insulin resistance.

We found improvement in insulin resistance measured by the HOMA-IR in both groups, but changes were more prominent after OAGB, which is in parallel with the findings of the YOMEGA study^59^. OAGB had a better success rate for T2DM (95%) in 1 year, even though patients with more serious diabetes underwent OAGB (40% of the patients were on insulin in the OAGB group vs none in the SG group). OAGB was superior in controlling T2DM which was consistent with weight loss, fasting resistin levels (especially after 3 months), and HOMA-IR changes.

The present study has two limitations. First, it had a nonrandomized design, as patients were assigned to either the OAGB or SG according to the patient-specific clinical criteria. Second, although we looked for hormone levels at different periods preoperatively and postoperatively, we analyzed fasting levels only, and we did not assess postprandial secretion of the selected hormones in response to a standardized meal test.

## Conclusions

The present study concludes that OAGB has better effects on weight loss and resolution of type 2 diabetes than SG. This is the first study to compare fasting ghrelin and resistin levels after OAGB compared to SG. Although similar changes were observed in the fasting levels of resistin and ghrelin, ghrelin changes were more prominent after SG, whereas resistin changes were more prominent after OAGB. OAGB was superior in the control of T2DM, which was in parallel with weight loss, fasting resistin levels, and HOMA-IR changes, suggesting a possible effect of resistin on glucose metabolism and insulin resistance.

## Ethical approval

The study protocol was approved by the Ethics Committee of Hacettepe University, Ankara (GO 14/203-18).

## Source of funding

This study was supported by the Turkish Scientific and Research Council (TUBITAK) through a grant (B.14.2.TBT.0.06.03.02-161-195358).

## Author contribution

M.M.O.: guarantor of integrity of the entire study; F.O., M.M.O., and T.T.Ş.: study concepts; F.O., T.T.Ş., and M.M.O.: study design and definition of intellectual content; F.O. and T. T.Ş.: literature research; F.O., T.T.Ş., A.D., and M.M.O.: data acquisition; M.M.O., T.T.Ş., and F.Ö.: clinical studies; F.Ö., A.D., and T.T.Ş.; data analysis; A.D., F.O., and T.T.Ş.: statistical analysis; F.O., M.M.O., T.T.Ş.: manuscript preparation; M.M.O., F.O., and T.T.Ş.: manuscript editing; M.M.O. and F.Ö.: manuscript review.

## Conflicts of interest disclosure

The authors declare that they have no affiliations with or involvement in any organization or entity with any financial interest in the subject matter or materials discussed in this manuscript.

## Research registration unique identifying number (UIN)

1. Name of the registry: Research Registry.
2. Unique identifying number or registration ID: 10054.
3. Hyperlink to the registration (must be publicly accessible): https://www.researchregistry.com/browse-the-registry#home/

## Guarantor

M. Mahir Ozmen.

## Data availability statement

It has been listed below each figure and table.

## Provenance and peer review

Not commissioned, externally peer-reviewed.

Acknowledgments
